# NMDA Receptor-Dependent Metaplasticity by High-Frequency Magnetic Stimulation

**DOI:** 10.1155/2014/684238

**Published:** 2014-10-28

**Authors:** Tursonjan Tokay, Timo Kirschstein, Marco Rohde, Volker Zschorlich, Rüdiger Köhling

**Affiliations:** ^1^Oscar Langendorff Institute of Physiology, University of Rostock, Gertrudenstraße 9, 18057 Rostock, Germany; ^2^Center for Life Sciences, Nazarbayev University, 53 Kabanbay Batyr Avenue, Astana 010000, Kazakhstan; ^3^Institute of Sport Sciences, University of Rostock, Ulmenstraße 69, 18057 Rostock, Germany

## Abstract

High-frequency magnetic stimulation (HFMS) can elicit N-methyl-D-aspartate (NMDA) receptor-dependent long-term potentiation (LTP) at Schaffer collateral-CA1 pyramidal cell synapses. Here, we investigated the priming effect of HFMS on the subsequent magnitude of electrically induced LTP in the CA1 region of rat hippocampal slices using field excitatory postsynaptic potential (fEPSP) recordings. In control slices, electrical high-frequency conditioning stimulation (CS) could reliably induce LTP. In contrast, the same CS protocol resulted in long-term depression when HFMS was delivered to the slice 30 min prior to the electrical stimulation. HFMS-priming was diminished when applied in the presence of the metabotropic glutamate receptor antagonists (RS)-*α*-methylserine-O-phosphate (MSOP) and (RS)-*α*-methyl-4-carboxyphenylglycine (MCPG). Moreover, when HFMS was delivered in the presence of the NMDA receptor-antagonist D-2-amino-5-phosphonovalerate (50 *µ*M), CS-induced electrical LTP was again as high as under control conditions in slices without priming. These results demonstrate that HFMS significantly reduced the propensity of subsequent electrical LTP and show that both metabotropic glutamate and NMDA receptor activation were involved in this form of HFMS-induced metaplasticity.

## 1. Introduction

High-frequency neuronal activity can induce persistent changes in synaptic strength resulting in a long-lasting increase of the postsynaptic response called long-term potentiation or LTP [1]. Hence, synaptic plasticity is comprised of both LTP and its counterpart, termed long-term depression (LTD) which is the long-lasting decrease of synaptic strength [2]. Since its discovery in the hippocampus [3], increasing evidence has suggested synaptic plasticity to be the basic mechanism of information storage in the brain. One major mechanism of LTP is the activation of postsynaptic NMDA receptors. Thus, it is currently accepted that electrical high-frequency stimulation of afferent fibers results in excessive glutamate release and subsequent AMPA receptor-mediated depolarization of the postsynaptic membrane which enables relief from the Mg^2+^ block [4]. NMDA receptor activation, in turn, leads to Ca^2+^ influx and thus activation of a number of metabolic processes resulting in a long-lasting enhancement of AMPA receptor-mediated responses. Initially, mechanisms of LTP induction were commonly studied in naive synapses, but later it was found that the magnitude of NMDA receptor-mediated LTP itself was subjected to prior neuronal activity. Weak stimulation or priming of afferent fibers that was insufficient to induce long-lasting changes by itself was able to significantly inhibit subsequent LTP induction, and this process—called metaplasticity—was also NMDA receptor-dependent [5]. Now, metaplasticity is regarded as a form of homeostasis in which the history of previous neuronal activation influences the direction and degree of synaptic plasticity elicited by a given stimulus [6].

Synaptic plasticity cannot only be induced by electrical stimulation. In vivo studies in humans using repetitive transcranial magnetic stimulation (rTMS) have indicated persistent changes in various outcome measures that have been referred to as LTP- or LTD-like changes [7]. In addition, we recently applied high-frequency magnetic stimulation (HFMS) to hippocampal slices in vitro and were able to identify an NMDA receptor-dependent form of LTP [8]. Therefore, we hypothesized that HFMS might also change the propensity to express synaptic plasticity in response to subsequent electrical stimulation. In the present study, we have observed that HFMS priming indeed inhibited subsequent LTP induction by electrical stimulation of afferent fibers in the CA1 region. Moreover, the HFMS priming effect turned out to be NMDA receptor-dependent.

## 2. Materials and Methods

### 2.1. Slice Preparation

Hippocampal slices were prepared using 2- to 3-month-old male CD rats (Charles River Laboratories, Sulzfeld, Germany). All experiments conformed to local (German Animal Welfare Act) and international (European Council Directive 86/609/EEC) guidelines on the ethical use of animals. All efforts were made to minimize animal suffering and to reduce the number of animals used. After deep anesthesia with diethyl ether, rats were decapitated and the brain was rapidly removed and submerged into oxygenated ice-cold dissection solution containing (in mM) 125 NaCl, 26 NaHCO_3_, 3 KCl, 1.25 NaH_2_PO_4_, 0.2 CaCl_2_, 5 MgCl_2_, and 13 D-glucose (95% O_2_, 5% CO_2_; pH 7.4; Osm 306–314 mosmol/kg). Horizontal brain slices (400 *μ*m) of the hippocampus were prepared using a vibratome (Campden Instruments, Loughborough, UK), and slices were then transferred into a holding chamber containing artificial cerebrospinal fluid (ACSF) containing (in mM) 125 NaCl, 26 NaHCO_3_, 3 KCl, 1.25 NaH_2_PO_4_, 2.5 CaCl_2_, 1.3 MgCl_2_, and 13 D-glucose (Osm 306–314 mosmol/kg). Slices were continuously bubbled with 95% O_2_ and 5% CO_2_ to maintain the pH at 7.4 and were allowed to recover at room temperature (20–22°C) for at least 1 hour before being transferred into recording chamber.

### 2.2. Electrophysiological Recording and LTP Induction

Hippocampal slices were transferred into an interface chamber and continuously superfused with oxygenated ACSF at flow rate of 2-3 mL/min with a volumetric infusion pump MCM-500 (MC Medicine technique GmbH, Alzenau, Germany) and the solution temperature was controlled at 32 ± 1°C by (npi electronic GmbH, Tamm, Germany). The experiments started after an equilibration time of at least 30 min. Field excitatory postsynaptic potentials (fEPSPs) were recorded using borosilicate glass pipettes (2-3 MΩ, pulled with PIP5 from HEKA Electronik, Lambrecht, Germany) filled with ACSF. Stimulating and recording electrodes were placed into CA1 stratum radiatum. Bipolar stimulation was performed with a platinum wire electrode and applied to Schaffer collaterals with ISO-STIM01M stimulus isolator (npi electronic GmbH, Tamm, Germany) controlled by a Master-8 stimulator (A.M.P.I., Jerusalem, Israel). Recording signals were amplified and filtered at 1 kHz by an EXT-10-2F (npi electronic GmbH, Tamm, Germany). Analog data were digitized with a Micro1401 analog-to-digital converter (Cambridge Electronic Design, Cambridge, UK) and stored for offline analysis using Signal 2.16 software (Cambridge Electronic Design, Cambridge, UK). Short-term plasticity was evaluated using paired-pulse stimulation (interstimulus intervals 20–500 ms). The paired-pulse ratio (PPR) was calculated as the 2nd fEPSP amplitude divided by the 1st fEPSP amplitude. For long-term potentiation (LTP) experiments, the Schaffer collateral pathway was stimulated at a rate of 0.033 Hz with the baseline stimulation strength adjusted to 30–40% of the maximal fEPSP amplitude (using paired-pulse stimulation with interstimulus interval 40 ms). LTP was induced with a conditioning stimulation (CS) protocol consisting of 10 bursts (1 s apart) of 20 pulses at 100 Hz (double baseline stimulation strength). Following CS, fEPSPs were continuously recorded for another 60 min after CS, and LTP was evaluated as the fEPSP slope at 60 min after CS delivery as the percentage of the baseline fEPSP slope. The specific NMDA receptor antagonist D-2-amino-5-phosphonopentanoate (D-AP5) as well as the metabotropic glutamate receptor antagonists (RS)-*α*-methylserine-O-phosphate (MSOP, group III antagonist) and (RS)-*α*-methyl-4-carboxyphenylglycine (MCPG, group I/II antagonist) were purchased from Tocris (Bristol, UK). All other chemicals used for physiological solutions were purchased from Sigma (Taufkirchen, Germany).

### 2.3. High-Frequency Magnetic Stimulation

High-frequency magnetic stimulation (HFMS) was performed using a magnetic stimulator MagPro R100 (Medtronic, Skovlunde, Denmark) equipped with a circular coil (type MC-125; diameter = 130 mm, thickness = 11.3 mm). The HFMS protocol consisted of 10 bursts (1 s apart) of 20 pulses at 100 Hz with 5 repetitions (10 s apart) as previously published [8]. HFMS was delivered to hippocampal slices through the coil positioned vertically and closely above the surface of the slices (8 mm distance). The intensity of magnetic stimulation was adjusted to 40–50% of its maximal output in all slices (corresponding to 60–75 A/*μ*s).

### 2.4. Statistical Analysis

All data are expressed as mean values and the standard error of the mean. Statistical comparison was performed using Student's two-tailed *t*-test or two-way ANOVA as indicated. Significant differences were indicated with asterisks in all figures (^*^ *P* < 0.05, ^**^ *P* < 0.01, and ^***^ *P* < 0.001).

## 3. Results

### 3.1. Magnetic Stimulation Inhibits Subsequent LTP

**Figure 1:**
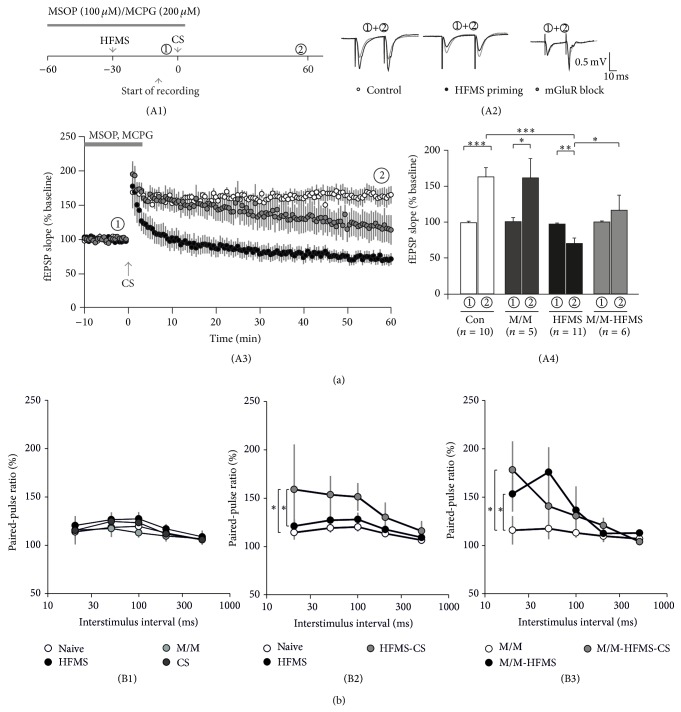
Electrical stimulation-induced LTP is lost after HFMS priming. (A1) Experimental paradigm showing the timepoints of high-frequency magnetic stimulation (HFMS) and conditioning stimulation (CS). Application of the mGluR blockers MSOP and MCPG is indicated by the gray bar. (A2) Sample traces of timepoints ① and ② in naive slices (control), in HFMS-primed slices, and under mGluR-blocking conditions. (A3) Time course of electrical stimulation-induced plasticity with (closed circles) or without (open circles) priming with high-frequency magnetic stimulation (HFMS). A subset of slices was bathed in MSOP and MCPG during HFMS and CS (gray circles). Baseline recording was started 20 min after priming stimulation using the following HFMS paradigm: 10 bursts (1 s apart) of 20 pulses at 100 Hz with 5 repetitions (10 s apart) as previously published [8]. At 30 min after HFMS (i.e., after 10 min baseline recording), slices were electrically stimulated (conditioning stimulation, CS) using a paradigm composed of 10 trains of 20 pulses at 100 Hz (1 s apart) at time point 0 min (gray arrow). (A4) Percentage of mean fEPSP slopes calculated during 56–60 min after CS application. Significant LTP was induced by the CS paradigm in control slices without priming stimulation (open bar). In contrast, a significant LTD, but not LTP, was induced in slices with priming HFMS (closed bar). MSOP/MCPG application did not alter LTP in naive slices (M/M) but prevented CS-induced LTD in HFMS-primed slices (M/M-HFMS). (b) Paired-pulse plasticity in control experiments (B1), metaplasticity experiments in standard solution (B2), and metaplasticity experiments in mGluR-blocking conditions (B3).

The aim of this study was to explore whether or not magnetic stimulation interferes with electrically induced LTP. To this end, we first applied high-frequency magnetic stimulation (HFMS) to the hippocampal brain slice, removed the magnetic coil, and then started electrophysiological recordings of the Schaffer collateral-CA1 synapse. The time schedule of this experiment is depicted in Figure 1(a)(A1). After a stable baseline was established, we delivered a conditioning stimulation (CS) paradigm to the Schaffer collaterals. Under control conditions, this protocol induced robust LTP at 60 min after CS (163 ± 13%, *n* = 10, open symbols/bars in Figure 1(a)(A3/4)). The paired-pulse ratio (PPR) did not change significantly at 60 min after CS indicating postsynaptic expression of LTP (100 ± 2% of baseline PPR, *n* = 10). To test for HFMS priming effects, a separate set of slices underwent magnetic stimulation as previously published [8] and were allowed to recover 20 min before electrophysiological recording was started. Following 10 min baseline recording, CS was applied as in unprimed control slices. In marked contrast to controls, slices that had experienced HFMS 30 min prior to electrophysiological recording showed significant LTD instead of LTP following the same CS protocol (70 ± 8%, *n* = 11, closed symbols/bars in Figure 1(a)(A3/4), *P* < 0.001 versus unprimed slices, unpaired *t*-test). Moreover, the paired-pulse ratio was significantly higher at 60 min after CS compared to baseline (114 ± 5% of baseline PPR, *n* = 11, *P* < 0.01 versus baseline, paired *t*-test) suggesting a presynaptic contribution to CS-induced LTD in HFMS-primed slices. Since both pre- and postsynaptic metabotropic glutamate receptors (mGluRs) might have been activated by excessive glutamate release following HFMS, we aimed to control for mGluR contribution to both the priming effect and the altered paired-pulse ratio. To this aim, we blocked both group I/II mGluRs using (RS)-*α*-methyl-4-carboxyphenylglycine (MCPG, 200 *μ*M) and group III mGluRs using (RS)-*α*-methylserine-O-phosphate (MSOP, 100 *μ*M). In naive slices, these blockers did not change the fEPSP amplitude (103 ± 14%, *n* = 10) or the paired-pulse ratio (102 ± 3%, *n* = 10). Moreover, LTP induction was also preserved in the presence of both mGluR blockers (161 ± 27%, *n* = 5, Figure 1(a)(A4)). However, the HFMS priming effect was significantly diminished in ACSF containing both mGluR blockers. In these experiments, LTD was prevented and the fEPSP slope at 60 min after CS was significantly higher than in HFMS-primed slices in standard bath solution (116 ± 21%, *n* = 6, *P* < 0.05, unpaired *t*-test, gray symbols/bars in Figure 1(a)(A3/4)). Unexpectedly, the paired-pulse ratio increased in these recordings (122 ± 7%, *n* = 6, *P* < 0.05 versus baseline, paired *t*-test; see also sample trace in Figure 1(a)(A2)). This prompted us to evaluate paired-pulse plasticity with varying interstimulus intervals (20–500 ms). First, we confirmed that the paired-pulse ratio was not altered by HFMS itself, MSOP/MCPG treatment, or CS delivery (Figure 1(b)(B1)). Intriguingly, HFMS priming and subsequent CS increased the PPR significantly over a wide range of interstimulus intervals (gray symbols in Figure 1(b)(B2), *P* < 0.05 versus naive and versus HFMS-primed slices, two-way ANOVA). To explore whether mGluRs were activated during HFMS priming, we again coapplied MSOP and MCPG prior to HFMS. Under these conditions, HFMS priming per se caused a significant increase of the PPR (closed symbols in Figure 1(b)(B3), *P* < 0.05 versus MSOP/MCPG-treated slices, two-way ANOVA), which was not further altered by subsequent CS (gray symbols in Figure 1(b)(B3)). These experiments clearly demonstrate that HFMS priming activates mGluRs which in turn depress paired-pulse plasticity and contribute to subsequent LTP suppression.

**Figure 2:**
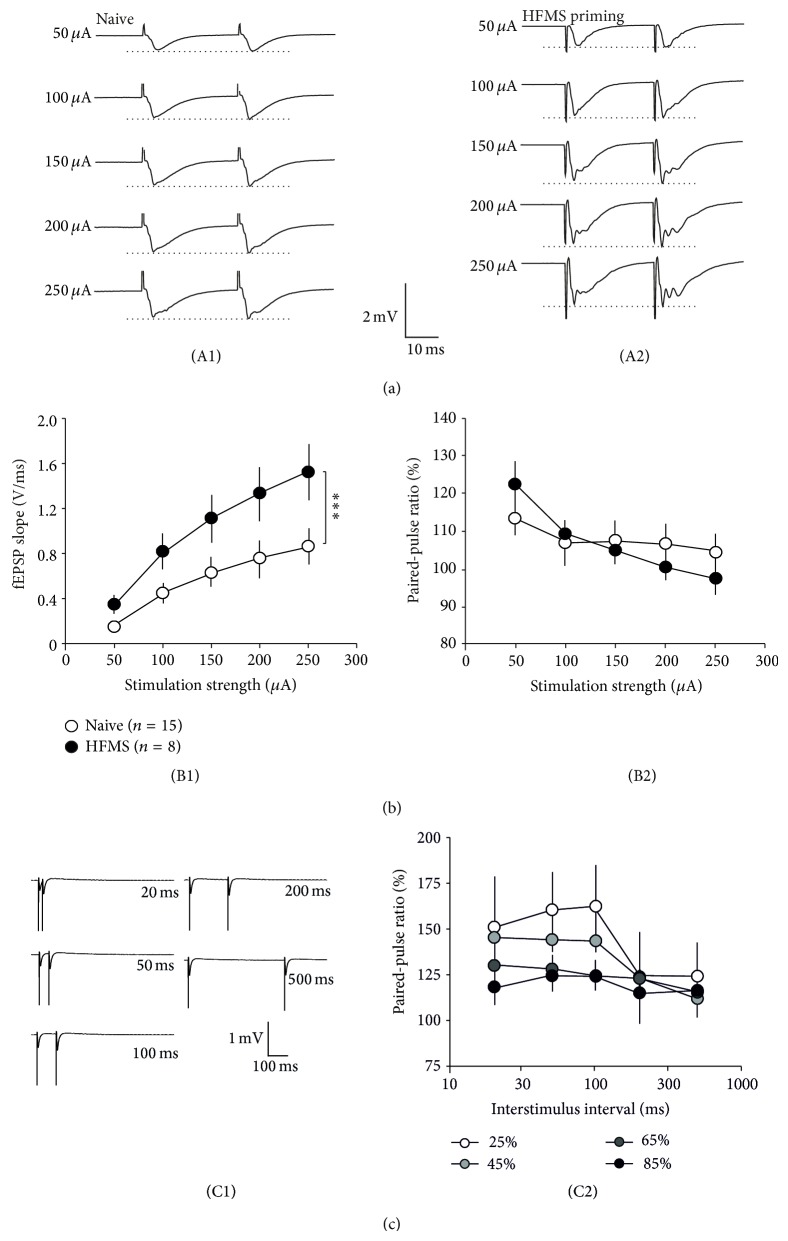
(a) Sample traces (interstimulus interval 40 ms) at increasing stimulation strengths (50–250 *μ*A) of naive (A1) and HFMS-primed slices (A2). Note the difference in amplitude between both representative recordings. (B1) Input-output curve of naive and HFMS-primed slices were significantly different. (B2) The paired-pulse ratio dropped significantly with increasing stimulation strength but was not different between naive and HFMS-primed slices. (C1) Sample traces of paired-pulse recordings at increasing interstimulus intervals (20–500 ms), obtained by stimulation strength adjusted to yield 45% of maximal fEPSP amplitude. (C2) The paired-pulse ratio is a function of stimulation intensity. The stimulation strength was adjusted to achieve 25%, 45%, 65%, or 85% of the maximal fEPSP amplitude.

However, these experiments so far do not explain why the paired-pulse ratio is enhanced by LTP induction only if delivered to prior HFMS (compare Figure 1(b)(B2) and Figure 1(b)(B1)). In this experimental paradigm, the input-output relationship has been determined at synapses which might have undergone potentiation by magnetic stimulation. We, therefore, obtained input-output curves from naive and HFMS-primed slices (Figure 2(a)(A1/2)) which showed a significant potentiation due to the magnetic stimulation (*P* < 0.001, two-way ANOVA; Figure 2(b)(B1)). From these data, we also calculated the paired-pulse ratio and observed that the PPR decreased significantly with increasing stimulation strength in both naive and HFMS-primed slices (*P* < 0.001, two-way ANOVA), while there was no significant difference between these two groups (*P* = 0.813, two-way ANOVA; Figure 2(b)(B2)). With respect to paired-pulse ratio and thus presynaptic transmitter release probability, it is likely that CS delivered to potentiated synapses is different from CS delivered to naive synapses. To test the effect of transmitter release on the paired-pulse ratio directly, we repeated our paired-pulse plasticity experiments with increasing stimulation strength.  Figure 2(c)(C1) shows an example of these recordings with a stimulation strength adjusted to achieve 45% of the maximal fEPSP amplitude. Reducing the stimulation strength to yield 25% of the maximal amplitude increased the PPR, while higher stimulus intensities decreased the PPR significantly (*P* < 0.001, two-way ANOVA; Figure 2(c)(C2)).

**Figure 3:**
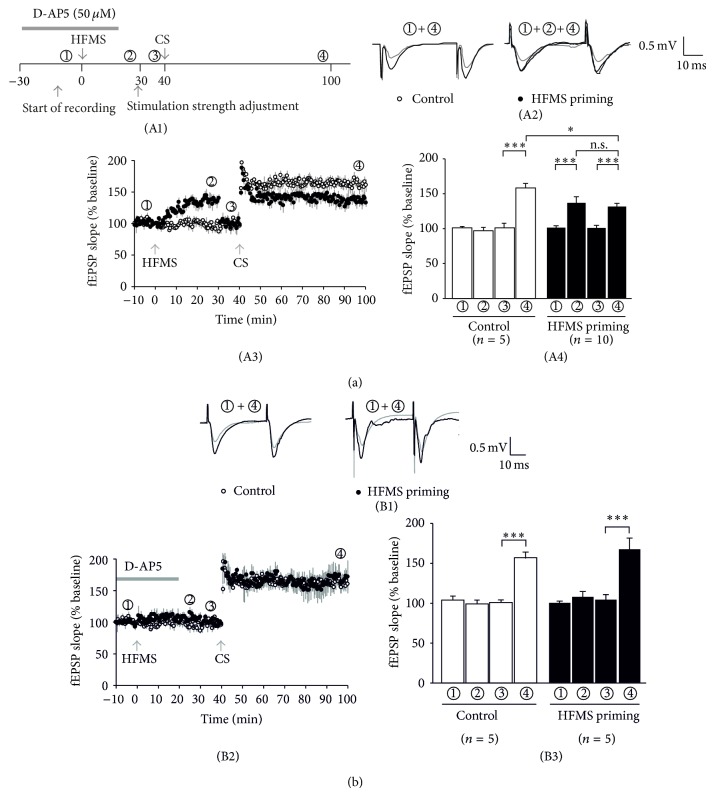
LTP reduction caused by HFMS priming is NMDA receptor-dependent. (A1) Experimental paradigm showing the timepoints of high-frequency magnetic stimulation (HFMS) and conditioning stimulation (CS). Application of the NMDA receptor blocker D-AP5 is indicated by the gray bar. (A2) Sample traces of timepoints ①, ②, and ④ in naive (control) and HFMS-primed slices. (A3) Time course of CS-induced plasticity with (closed circles) or without (open circles) priming. HFMS priming was applied following baseline recording. The enhanced fEPSPs by HFMS priming were readjusted to baseline level before electrical stimulation (CS). HFMS caused a significant reduction of CS-induced LTP. (A4) Average fEPSP slopes calculated during 26–30 and 96–100 min after HFMS application. Significant LTP was induced after CS in control slices without priming stimulation (open bar). In contrast, the level of CS-induced LTP in HFMS-primed slices was significantly reduced compared to slices without priming stimulation. Note that HFMS-induced potentiation and CS-induced potentiation were not significantly different. (B1) Sample traces of timepoints ① and ④ in naive (control) and HFMS-primed slices under NMDA receptor-blocking conditions. (B2) Time course of CS-induced plasticity with (closed circles) or without (open circles) priming. HFMS priming was applied in the presence of the NMDA receptor antagonist D-AP5 (50 *μ*M). The slightly enhanced fEPSPs following HFMS priming were readjusted to baseline level before electrical CS. D-AP5 was washed out after HFMS in order to allow NMDA receptor-dependent CS-induced LTP. (B3) Average fEPSP slopes calculated during 26–30 and 96–100 min after HFMS application. In contrast to panel (A4), the levels of CS-induced LTP were no longer different between HFMS-primed or unprimed slices.

These experiments predict that adjusting the baseline stimulation strength following HFMS-induced priming should preserve paired-pulse plasticity. Hence, we next carried out a series of experiments where the electrophysiological recording was started before HFMS in order to monitor changes in synaptic strength by magnetic stimulation (time schedule in Figure 3(a)(A1)). Indeed, we observed that in slices with HFMS priming (closed symbols in Figure 3(a)(A3)) the fEPSP slope was significantly enhanced after magnetic stimulation (138 ± 8%, *n* = 10, timepoint ② in Figure 3(a)(A3)). Therefore, we reduced the stimulation strength in order to obtain the same amplitude as at the beginning of the experiment (timepoint ③ in Figure 3(a)(A3)). Then, CS was delivered to the HFMS-primed slices and resulted in significant LTP of the fEPSP slope (133 ± 5%, *n* = 10, timepoint ④ in Figure 3(a)(A3)). Importantly, the magnitude of LTP in primed slices was significantly lower than in unprimed slices (*P* < 0.05, unpaired *t*-test; Figure 3(a)(A4)). In these unprimed control slices, the magnetic coil was also placed above the slice, but magnetic stimulation was omitted. In line with our previous data, LTP by CS in control slices showed the same magnitude as in the first set of experiments (164 ± 9%, *n* = 5, open symbols/bars in Figure 3(a)(A3/4)). Importantly, the paired-pulse ratio remained stable in these experiments (primed: 101 ± 3% of baseline PPR, *n* = 7; unprimed: 107 ± 5% of baseline PPR, *n* = 5). Thus, under these conditions, LTP induction no longer altered the paired-pulse ratio as in unprimed slices. These experiments suggest that CS per se does not lead to PPR changes, which is consistent with a postsynaptic mechanism of CS-induced LTP.

### 3.2. HFMS-Induced Metaplasticity Is NMDA Receptor-Dependent

Our results so far indicate that HFMS was able to reduce the propensity of subsequent LTP induction. Activation of mGluRs during HFMS appeared to contribute to this effect, but mGluR inhibition did not fully restore LTP. On the other hand, priming-induced LTP suppression, termed metaplasticity, may also involve NMDA receptors [5]. We therefore hypothesized that NMDA receptor inhibition during HFMS should restore the LTP magnitude to control level. To test this hypothesis, we applied the NMDA receptor blocker D-AP5 (50 *μ*M) during HFMS and observed that the fEPSP slope was still enhanced under these conditions, but the increase was not significant (105 ± 4%, *n* = 5, timepoint ② in Figure 3(b)(B2)). Nevertheless, the stimulation strength was also adjusted to reveal the same amplitude as at the beginning of the experiment (timepoint ③ in Figure 3(b)(B2)), before CS was delivered to the slice. Following HFMS under NMDA receptor inhibition, LTP was indeed restored to control levels (170 ± 9%, *n* = 5, closed symbols/bars in Figure 3(b)(B3)), as demonstrated by interleaved slices without HFMS priming (153 ± 8%, *n* = 5, open symbols/bars in Figure 3(b)(B3)). Both LTP values were no longer significantly different (Figure 3(b)(B3)). Hence, the HFMS priming effect was an NMDA receptor-dependent process.

## 4. Discussion

Transcranial magnetic stimulation (TMS) is commonly employed in the clinical setting since it allows for evoking muscle contractions using a noninvasive stimulation of the motor cortex in awake humans [9]. More recently, repetitive TMS (rTMS) has attracted scientific attention because it may lead to persistent changes in cortical excitability for hours beyond rTMS application [7, 10]. This rTMS-induced neural plasticity shares a number of features from long-term potentiation (LTP) and long-term depression (LTD) obtained from in vitro preparations. For instance, paired associative stimulation of the primary motor cortex by TMS and of the contralateral median nerve by conventional electrical stimulation-induced LTP- or LTD-like changes of the motor-evoked potential depending on the interstimulus interval which resembled spike-timing plasticity [11]. On the other hand, various protocols of high-frequency magnetic stimulation (HFMS) have been applied in vitro and found to induce long-lasting changes in synaptic strength [8, 12–14]. One major advantage of this approach is that in vitro preparations offer the opportunity to study pharmacology. We have previously shown that HFMS-induced LTP is NMDA receptor-dependent and thus a postsynaptic process, while the fiber volley was left unaltered arguing against an axonal change in excitability [8].

In the present study, we have investigated the effect of high-frequency magnetic stimulation (HFMS) on subsequent electrically induced LTP. We observed that LTP by electrical stimulation was impaired by prior HFMS and that this inhibition was prevented by NMDA receptor blockade. This effect—plasticity of subsequent plasticity or metaplasticity—may prevent synapses from saturating potentiation or depression and is hence regarded as a form of synaptic homeostasis [6]. In the first set of experiments, we applied HFMS and subsequently started electrophysiological recording. We found that electrical stimulation (called conditioning stimulation, CS) induced LTD rather than LTP when slices had experienced HFMS before. This is consistent with the idea of homeostatic metaplasticity formalized by the Bienenstock-Cooper-Munro (BCM) theory of bidirectional synaptic plasticity [15]. In this theory, the synaptic modification threshold (*θ*_*M*_), that is, the threshold for induction of LTP versus LTD, is subject to prior postsynaptic activity. Thus, recent postsynaptic activity such as HFMS-induced potentiation should increase *θ*_*M*_ and favor the propensity of LTD induction. In fact, we found that the same CS protocol which induced robust LTP in control slices led to significant LTD in HFMS-primed slices. However, one might argue that pre-CS stimulation strength was quite high in these experiments because recordings were started after HFMS. Moreover, the paired-pulse ratio dropped significantly during LTD expression in HFMS-primed slices suggesting also presynaptic contribution under these conditions. Indeed, we could confirm that paired-pulse plasticity is a function of stimulation strength, and when HFMS priming was monitored in order to adjust pre-CS stimulation strength, the paired-pulse ratios remained stable in both primed and unprimed groups suggesting a predominantly postsynaptic nature of LTP expression. In these experiments, CS still induced LTP in HFMS-primed slices, but significantly less than in unprimed control slices. We conclude from these data that HFMS shifted *θ*_*M*_ to higher values and that this *θ*_*M*_ increase was even more pronounced in HFMS-potentiated synapses.

What does magnetic stimulation do? Along with Faraday's principle, alternating magnetic stimulation is thought to lead to electrical currents in conductive tissues. Alternating currents cause membrane potential changes, depending on their intrinsic properties such as the membrane time constant. Assuming that this ultimately leads to glutamate release, glutamate receptors should be activated by magnetic stimulation. We have previously shown that NMDA receptors are activated via HFMS leading to synaptic potentiation [8]. Now, we add the important information that HFMS leads to metaplasticity involving both mGluRs and NMDARs. What are the mechanisms of HFMS-induced metaplasticity? Currently, homosynaptic metaplasticity is believed to follow either activation of group I metabotropic glutamate receptors which facilitates and prolongs LTP [16] or activation of NMDA receptors which inhibits subsequent LTP [5] and facilitates LTD [17]. In our hands, the HFMS priming effect on subsequent LTP was significantly diminished by mGluR inhibition suggesting that mGluR activation during HFMS contributed to the LTP suppression. On the other hand, NMDA receptor inhibition itself was sufficient to prevent LTP suppression following prior HFMS indicating a predominantly postsynaptic mechanism of magnetic stimulation-induced metaplasticity. Mechanistically, it is likely that NMDAR-mediated metaplasticity involves Ca^2+^ influx and subsequent activation of signal transduction enzymes such as Ca^2+^/calmodulin-dependent kinase II (CaMKII). In the dentate gyrus for instance, it was found that NMDAR-dependent priming involved CaMKII phosphorylation at inhibitory sites [18]. In summary, our study has demonstrated that magnetic stimulation in vitro can influence subsequent electrically induced synaptic plasticity involving both mGluR and NMDAR activation.
